# Misconceptions and traditional practices towards infant teething symptoms among mothers in Southwest Ethiopia

**DOI:** 10.1186/s12903-018-0619-y

**Published:** 2018-09-21

**Authors:** Addis Getaneh, Fikirte Derseh, Michael Abreha, Tewodros Yirtaw

**Affiliations:** 1 Department of Dental and Maxillofacial, St.Paul Hospital’s Millennium Medical College, Addis Ababa, Ethiopia; 2 Department of Dentistry, Jimma University, Jimma, Ethiopia; 3 Department of Public Health, St. Paul Hospital’s Millennium Medical College, Addis Ababa, Ethiopia

**Keywords:** Teeth eruption, Symptoms, Infant, Culture, Ethiopia

## Abstract

**Background:**

The assumption of a link between common symptoms such as febrile illness, diarrhea and the eruption of primary teeth has been established over many centuries. According to traditional beliefs in Ethiopia, diarrhea and fever at the time of milk teeth eruption may be due to a worm in the child’s gums. Current medical observations show little more than restlessness, drooling, and finger sucking resulting from teething. The purpose of this research was to assess mothers’ traditional beliefs and practices towards teething symptoms.

**Methods:**

A cross-sectional descriptive study design was used with the convenience sampling technique. Mothers were approached at the pediatric Out Patient Department (OPD) of Jimma University Specialized Hospital, southwest Ethiopia. A structured questionnaire was used for data collection. The data were analyzed by SPSS (version 20).

**Results:**

A total of 107 mothers were interviewed. Ninety-eight (91.6%) claimed that teething was associated with various symptoms. Ninety-seven (90.7%) attributed diarrhea to teething. Only one mother said she would give her child Paracetamol to relieve the teething symptoms. Five (4.7%) mothers said they would allow their children to bite on a pacifier. Ten mothers (9.3%) said that they would prefer the child’s milk tooth to be extracted. Some of the practices by mothers to relieve the symptoms include rubbing the gum of the child with garlic (12.1%) or rubbing the gum with herbs (6.5%).

**Conclusions:**

Most of the mothers had misconceptions about the symptoms that usually appear during teething. Health education should be provided by dentists and professionals concerned with child care in correcting these misconceptions and cultural beliefs about teething symptoms.

**Electronic supplementary material:**

The online version of this article (10.1186/s12903-018-0619-y) contains supplementary material, which is available to authorized users.

## Background

The eruption of teeth (teething) is the movement of the teeth from their pre-eruptive position in the alveolar bone through the mucosa into the oral cavity as defined by Carpenter J V in 1978 in a study that assessed the relationship between teething and systemic disturbance [1].Teething usually begins around 6 months and lasts until approximately 3 years of age [2]. Most parents regard the eruption of an infant’s first tooth as a significant developmental landmark, and an ‘old wives’ tale’ regards its precocious eruption as a sign of great intelligence. The relationship between the eruption of the primary teeth and the general health of infants has been held for over 5000 years [3]. A cross-sectional study of five groups of child health professionals versus parents and medical personnel in Australia and Israel, respectively, indicates that these assumptions are not held by parents alone [4, 5]. The issue of symptoms associated with teething had been debatable with some authors asserting that different symptoms are associated with teething while others claimed the contrary [3, 4, 6, 7]. Traditional beliefs also strongly associate specific symptoms with times of teething. Some of the symptoms that had been associated with teething in children include fever, diarrhea, general irritability, drooling of saliva, sleep disturbance and ear infection [5]. Others include pain, inflammation of the mucosa overlying the tooth, facial flushing, circumoral rash, gum rubbing (biting), sucking, constipation, and loss of appetite [4, 8] Traditional beliefs in Uganda assert that primary canines are sources of illness and should be extracted to prevent the illness [9]. According to traditional beliefs in Ethiopia, diarrhea and fever at the time of milk teeth eruption may be due to a worm in the child’s gum [10]. The National Baseline Survey on Harmful Traditional Practices (HTPs) of Ethiopia carried out in 1997 found that milk tooth extraction is performed in more than 80.2% of the general population. In the Gambella region, there is a very high prevalence of this tradition that is valued as a marker of ethnic identity in the region. In the Oromia region, the prevalence of this practice is 89% [10]. Current medical studies show little more than restlessness, drooling, finger sucking and appetite loss arising from teething [7, 11, 12]. The timing of eruption of the primary incisors (6–12 months) coincides with the decrease in the circulating maternal humoral immunity conferred via the placenta and the development of the child’s own humoral immunity. Consequently, children of this age are prone to myriad of relatively minor infections [12]. In Ethiopia, there is a misconception that symptoms associated with teething are relieved by having the teeth extracted or by having the gum drilled and the primary teeth or the new permanent teeth extracted or craved out [10]. The objective of this research was to assess mothers’ traditional beliefs and practices towards teething symptoms.

## Methods

### Study area

The study was conducted in Jimma University Specialized Hospital (JUSH) located in southwest Ethiopia, Oromia region. Jimma is the largest city in southwest Ethiopia located 352 km away from Addis Ababa. Jimma University Specialized Hospital is one of the oldest public hospitals in the country. It was established in 1937 by Italian invaders for the service of their soldiers. It is the only teaching and referral hospital in the southwestern part of the country. It provides services for approximately 15,000 inpatients, 160,000 outpatients, 11,000 emergency cases and 4500 deliveries a year from the catchment population of about 15 million people.

### Ethical consideration

The Informed consent process was approved by the Jimma University’s Institute of health sciences community-based education office (CBE). Permission was also obtained from the JUSH Medical director’s office. Verbal Informed consent of the participants was obtained. The confidentiality of the information they provide was assured by removing unique identifiers from the questionnaire. The privacy of the mothers was maintained during data collection as the interview was conducted in a secluded area.

### Study period

The data were collected from August 10–14, 2015.

### Study design

Cross-Sectional study design was used.

### Sample population

All mothers who visited JUSH’s pediatric OPD, southwest Ethiopia from August 10–14, 2015**.**

### Sample size and sampling technique

Convenience sampling technique was used. The working days of the week from August 10–14, 2015 were randomly selected. All mothers who presented at the pediatric OPD and verbally consented were interviewed.

### Data collection and analysis

Data were collected by the interviewer-administered questionnaire which was adapted from studies done by Kakatkar et al. in India and by Awadkamil in Sudan [8, 13]. Traditional practices identified by the National Baseline Survey on Harmful Traditional Practices of Ethiopia were included in the questions [10]. The questionnaire contains three items, the first part was to assess the mother’s knowledge about tooth eruption, the second was about the various symptoms attributed to teething, and third item was on practices the subjects preferred to relieve teething symptoms (Additional file 1). The questionnaire was developed in English (as English is the language of instruction of higher education in Ethiopia) but was translated to local languages of Amharic and Afaan Oromo by a native speaker who can speak both languages as well as proficient in English. A pre-test of the translated questionnaire was done on 11 mothers to test the clarity of the questions and their feedback was considered to improve the understandability of the questions.

Income classification used for this study is based on the scheme of Africa Development Bank (AfDB). In this study, it is adjusted to income per month and converted to Ethiopian currency ‘Birr’ according to National bank of Ethiopia currency rate during the data collection time.

**Poor class**: - those who earn less than 2$/day

**Floating class: -** those who earn 2$-4$/day

**Lower middle class: -** those who earn 4$-10$/day

**Upper middle class:** - those who earn 10$-20$/day

**Rich class: -** those who earn greater than 20$/day

Data collected were analyzed by SPSS software (version 20). Descriptive statistics were used. Chi-square tests of significance (Fisher’s exact test) were used with a 95% confidence interval (*p* < 0.05).

## Result

### Socio-demographic profile of the study population

A total of 107 mothers were interviewed. The mean age of the mothers was 29.07 (SD 9.24); Sixty-two (57.9%) were within the age range of 20–29. Sixteen (15%) of the mothers had no formal education, four (3.7%) were only able to read and write, and 24 (22.4%) had a university or college education. Fifty-three (49.5%) were housewives, civil servants were 24 (22.4%) and 6(5.6%) were professionals. Ninety-two (86.0%) live in urban. Thirty (28%) of the mothers comprised of the poor class while only Fourteen (8.4%) made up the rich class **(**Table 1**)**.

**Table 1:** Socio-Demographic profile of the study population

*Demographic characteristics*		*Number*	*%*
Age	< 20	4	3.7
	20–29	62	57.9
	30–39	35	32.7
	> 50	6	5.6
	Total	107	100.0
Education	Illiterate	16	15.0
	Red and write	4	3.7
	Primary	32	29.9
	Secondary	28	26.2
	Post-secondary	3	2.8
	University/college	24	22.4
	Total	107	100.0
Occupation	Farmer	4	3.7
	Civil servant	24	22.4
	Professional	6	5.6
	Merchant	15	14.0
	Housewife	53	49.5
	Other	5	4.7
	Total	107	100.0
Income Class	Poor class	30	28.0
	Floating class	22	20.6
	Lower middle class	26	24.3
	Upper middle class	20	18.7
	Rich class	9	8.4
	Total	107	100.0
Place of residence	Urban	92	86.0
	Rural	15	14.0
	Total	107	100.0

### Knowledge of the mothers about teeth eruption

Seventy (65.4%) of the mothers thought that teeth start to erupt around 6–7 months of age (Table 2) and it was statistically significant with their age (*p*-value = 0.031) (Table 3). While 80 (74.8%) of them believed that the lower central incisors appear first in the mouth and this differed with the place of residence (*p*-value = 0.006). Forty-one (38.3%) of them believed that teeth eruption ends around 2 years of age and it was statistically significant with educational level (*p*-value = 0.019) and place of residence (*p*-value = 0.002) (Table 3). 29(27.1%) believed that delayed eruption was associated with systemic disease **(**Table 2**).**

**Table 2:** Mothers’ knowledge about teething

Questions	Yes	No	Don’t know	Total
Babies’ teeth start to erupt around 6–7 months of Age	70(65.4%)	19(17.8%)	18(16.8%)	107(100%)
The first teeth to appear in the mouth are lower central incisors	80(74.8%)	21(19.6%)	6(5.6%)	107(100%)
The eruption of teeth gets completed at approximately 2 years of age	41(38.3%)	45(42.1%)	21(19.6%)	107(100%)
Delayed eruption of teeth may be an indication of the presence of systemic disease	29(27.1%)	74(69.2%)	4(3.7%)	107(100%)

**Table 3:** Association between knowledge items and socio-demographic profile of the mothers’

	Knowledge items			
Socio-demographic variables	Delayed eruption of teeth may be an indication of the presence of systemic disease Value(*P*-value)	Babies’ teeth start to erupt around 6–7 months of Age Value(*P*-value)	The first teeth to appear in the mouth are lower central incisors Value(*P*-value)	The eruption of teeth gets completed at approximately 2 years of age Value(*P*-value)
Age	3.33(0.776)	12.13(0.031)	4.14(0.629)	5.22(0.497)
Educational level	7.93(0.644)	9.45(0.424)	14.51(0.083)	18.99(0.019)
Place of residence	1.5(0.477)	2.75(0.286)	9.89(0.006€)	11.25(0.002)

Fisher’s exact test

### Perception of symptoms associated with teething

Most mothers (91.6%) perceived that teething was associated with various symptoms like diarrhea, fever, poor appetite, irritability, vomiting; while only 9 (8.4%) thought that teething was not associated with any symptoms. Most mothers (90.7%) attributed diarrhea, (72.9%) irritability, (61.7%) poor appetite, (48.6%) fever or vomiting (35.5%) to teething (Table 4). The mother’s association of fever with teething was statistically significant with age (*P*-value = .024) **(**Table 5**).**

**Table 4:** Symptoms ascribed to teething by mothers

Symptoms	Frequency (%)		Total
	Yes	No	
Fever	52(48.6%)	55(51.4%)	107(100%)
Diarrhea	97(90.7%)	10(9.3%)	107(100%)
Vomiting	38(35.5%)	69(64.5%)	107(100%)
Irritability	78(72.9%)	29(27.1%)	107(100%)
Poor appetite	66(61.7%)	41(38.3%)	107(100%)

*The numbers do not add up to 100 because of multiple symptoms by many of the mothers*

**Table 5:** Association between teething symptoms and socio-demographic characteristics

	Fever Value(*P*-value)	Diarrhea Value(*P*-value)	Vomiting Value(*P*-value)	Irritability Value(*P*-value)	Poor appetite Value(*P*-value)
Age	8.57(.024)	2.51(0.439)	2.014(0.585)	1.52(0.766)	1.71(0.66)
Educational level	3.53(0.645)	8.29(.092)	7.39(0.169)	9.21(0.077)	6.89(0.209)
Place of residence	0.268(0.159)	0.146(0.146)	0.248(0.143)	0.228(0.182)	0.570(0.329)

65.3% of the mothers said they would take their child to hospital during teething symptoms,which was not statistically significant with age, educational level and place of residence (Table 6 and Table 7). Only one mother (0.9%) said she would give her child Paracetamol during perceived teething symptoms whereas ten (9.3%) said they would want their child’s teeth to be extracted (Table 8) and it was statistically significant with the mother’s age (*p*-value = 0.008). There were 60 (56.1%) who said they would not give their child anything **(**Table 9**)**. 36.6% claimed that non-treated teething symptoms would result in severe illness, while 7.5% thought death could happen (Table 10).

**Table 6:** Mothers’ willingness to take their child to hospital

I will take the child to the hospital during teething	Frequency	Percent
Yes	64	65.3
No	34	34.7
Total	98	100.0

**Table 7:** Association between mothers’ willingness to take their child to the hospital and socio-demographic characteristics

	I will take to hospital Value(*P*-value)
Age	3.59(0.298)
Educational level	6.543(0.241)
Place of residence	0.584(0.39)

**Table 8:** Treatments provided by mothers during teething

To relieve pain	Frequency (%)
Paracetamol	1(0.9%)
Rubbing the gum with garlic	13(12.1%)
Giving pacifier	5(4.7%)
Herbs	7(6.5%)
Salt water	9(8.4%)
Extracting the teeth	10(9.3%)
Nothing	60(56.1%)

**Table 9:** Association between treatments of teething and socio-demographic characteristics

	Paracetamol	Rubbing the gum with garlic	Giving pacifier	Herbs	Salt water	Extraction	Nothing
Age	4.58 (0.421)	10.74 (0.008)	1.02 (0.788)	1.83 (0.630)	10.53 (0.01)	10.67 (0.008)	10.21 (0.01)
Education al level	6.65 (0.439)	6.75 (0.187)	5.74 (0.250)	2.29 (0.853)	11.003 (0.021)	3.77 (0.535)	10.02 (0.057)
Place of Residence	1.00 (0.86)	1.00 (0.576)	1.00 (0.463)	0.254 (0.254)	0.354 (0.242)	0.629 (0.424)	0.415 (0.273)

Fisher’s exact test: value (p-value)

**Table 10:** Mothers’ perception on outcome of non-treated teething symptoms

	Frequency	Percent
Poor growth	23	21.5
Severe illness	37	34.6
Nothing	39	36.4
Death	8	7.5
Total	107	100.0

## Discussion

Regarding the knowledge of mothers about tooth eruption in the current study, 65.4% believed that teeth eruption starts from 6 to 7 months of age and it differed according to mothers’ age(*p*-value = 0.031),which was less than the study conducted in India by Kakatkar et al. in 2010 (81.3%) [8]. 74.8% thought it was the lower incisors that erupt first, which was statistically significant with the mothers’ place of residence (*p*-value = 0.006) and was less than the study in India (81.3%) [8]. 38.3% believed that the entire set of primary teeth erupt at around 2 years of age, which was statistically significant with mothers’ educational level (*p*-value = 0.019) and place of residence (*p*-value = 0.002), which was significantly less than the study in India (76.3%) [8]. 27.1% of the mothers perceived delayed eruption as an indication of systemic disease which is higher than the study in India (44.7%) [8].The mothers knowledge in the Indian study [8] was significantly better than the current study.The reason for this difference may be partly due to better oral health education in India. In this study, most of the mothers perceived that teething is associated with various ailments (91.6%). This is comparable to a study in neighboring Sudan by Awadkamil in 2012, which reported 95% of study subjects to associate teething with different symptoms [13]. A Similar finding was also reported in Lagos State, Nigeria by Uti et al.*. in* 2005, where 95.2% of the mothers ascribed various ailments to teething [14]. However, another study in Ibadan, Nigeria reported a relatively lower percentage (64.8%) by Ige and Olubukola in 2013 [15].This may be related with the study design, sampling technique and cultural differences. In the study in Egypt 98.2% stated teething has associated symptoms (El-gilany et al.*.* in 2017) [16]. This difference may be partly due to the larger sample size of the study in Egypt. In the current study 90.7% of the mothers perceived diarrhea at the time of teeth eruption to be associated with teething, which is in agreement with the study in India (87.5%) [8], but less comparable with the study in Sudan (83.3%) [13]. In Ibadan, Nigeria 44.8% reported diarrhea to be associated with teething [15]. The other study in Nigerian Lagos state by *Uti* et al in 2005, found 64.0% to associate diarrhea to teething [14].In Egypt 51.0% reported diarrhea as one of the symptoms during teething [16].These differences across countries may be attributed to the difference in oral health literacy,study design and cultural diversity.Irritability was the second most attributed symptoms to teething in this study (72.9%), which is higher than that found in Ibadan, Nigeria (60.3%) [15] and Egypt (21.4%) [16], but lower than in Sudan (90.5%) [13]. In our study 61.7% of the mothers thought that teething caused poor appetite, which was lower than in Sudan (75%) [13], but higher than in Ibadan, Nigeria (48.6%) [15], Egypt (45.1%) [16],Lagos state, Nigeria (27.3%) [14],and India (23.5%) [8]. About half of the mothers (48.6%) believed that children would experience fever at the time of teething; this proportion is lower than in Nigeria Lagos state (80.5%) [14], Ibadan, Nigeria (82.1%) [15], Sudan (86.6%) [13], Egypt (83.2%) [16] and India (70%) [8]. This variability may be attributed to mother’s ability to report symptoms, which may be influenced by their knowledge, belief and health information. Vomiting was also one of the symptoms associated with teething by 35.5% of the mothers in the current study which agrees with the studies in Ibadan, Nigeria by Ige and Olubukola in 2013, which reported vomiting in (32.4%) [15], Lagos state Nigeria (32.8%) [15], India (37.1%) [8]. However, this figure was much higher than the study in Egypt by El-gilany et al. in 2017, where only 14.7% claimed vomiting to be associated with teething [16]. Since mothers associate these symptoms with teething they do not consider them as serious. 64.3% of mothers in this study said they would take their child to health facilities, which is much higher than in Sudan where only 16% of the mothers said they would want a doctor to see their child [13]. In India 86.4% of mothers would consult their primary health care provider in case problems arose during tooth eruption [8]. About half of the mothers (56.1%) said that they would not give their child anything at home during teething, which is lower than in Sudan (62%) [13]. Ten mothers (9.3%) said that they would want the child’s primary tooth to be extracted to stop the symptoms they associated with teething. The study in Sudan found that 14% the mothers did a procedure traditionally called ‘Haifat’ which is lancing of the gum and alveolar bone around the erupting primary canine [13]. Gum lancing was also stated by the study in Egypt(4.6%) [16]. In Uganda, a practice locally Known as ‘Ebino’, which is extraction of primary canines, was reported by Iriso et al. in 2000 [17]. 6.5% of the mothers perceived rubbing the gum with herbs as treatment; this is less comparable with the study in Egypt (9%) [16] and Sudan (11%) [13]. Only one mother said she would give her child paracetamol during teething, which is lower than in Sudan (9%) [13] and much lower than in Egypt in which 71.3% said they would give analgesics [16]. A Pacifier was the other teething remedy in this study(4.7%). This finding was much lower than in Egypt(31.3%) [16].The differences in mothers’ belief and practices about tooth eruption between the current study and the other literature used in the current study may be attributed to the study design and sampling,cultural diversity across countries,and oral health literacy.

## Conclusions

The study demonstrated that mothers included in this study still attribute various ailments to teething. Most of the mothers perceived diarrhea and fever to be associated with teething, which does not need medical attention. About half of the mothers did not know how to handle teething symptoms at home. There are also mothers who still prefer traditional practices like rubbing the gum with garlic and herbs, or extracting milk teeth.

## Limitation

The limitation of this study was the small sample size and non-probability sampling technique. The duration of the data collection was also short. Therefore, the findings of this study should be interpreted cautiously. But the study can serve as a baseline for future larger community based research.

## Recommendation

Health education should be given by dentists and pediatric health professionals to change the misconception and cultural beliefs about teething symptoms.

## Additional file

Additional file 1: Questionnaire. It is the data collection questionnaire that was used to interview the study participants. (DOCX 21 kb)

## Abbreviations

CBE: Community Based Education
HTP: Harmful Traditional Practices
JUSH: Jimma University Specialized Hospital
OPD: Out Patient Department
SPSS: Statistical Package for Social Science
