# Supplementary material for: Misconceptions and traditional practices towards infant teething symptoms among mothers in Southwest Ethiopia
Source: BMC Oral Health. 2018 Sep 21;18:159. doi: 10.1186/s12903-018-0619-y (PMC6151031; doi:10.1186/s12903-018-0619-y)
Supplement: Supplementary file 1 — Questionnaire. It is the data collection questionnaire that was used to interview the study participants. (DOCX 21 kb) [file 12903_2018_619_MOESM1_ESM.docx]

**Questionnaire**

All information given to us will be treated as strictly confidential and will not be made available to any other party. Your co-operation is highly appreciated. Please help us give your answers as accurate as possible.

1. 1) Name (optional)………………………………………………………………

2) Address………………………………………………………………………

3) Occupation……………………………………………………………………

4) Educational level…………………………………………………………….

5) Age ……………………………………………………………………………

6) Income ……………………………………………………………………….

1. Please answer these questions as accurately as you can.

1. Do you think that babies’ teeth start to erupt around 6–7 months of Age?

| Yes | No | Don’t know |
| --- | --- | --- |
|  |  |  |

2. Do you think that the first teeth to appear in the mouth are lower central incisors?

| Yes | No | NOT SURE |
| --- | --- | --- |
|  |  |  |

3. Do you think that the eruption of teeth gets completed at approximately 2 years of age?

| YES | NO | DON’T KNOW |
| --- | --- | --- |
|  |  |  |

4. Do you think that delayed eruption of teeth may be an indication of the presence of systemic disease?

| Yes | No | Don’t know |
| --- | --- | --- |
|  |  |  |

5. Do you worry about the time your baby’s teeth start to erupt?

| YES | NO | NOT BOTHERED |
| --- | --- | --- |
|  |  |  |

6. Do you think babies have any problems when their teeth are erupting?

| YES | NO |
| --- | --- |
|  |  |

7. What symptoms do you think are associated with teething in children?

| FEVER | LOOSE STOOLS | VOMITING | POOR  APPETITE | COUGH | UNDUE  CRYING | ANY  OTHER |
| --- | --- | --- | --- | --- | --- | --- |
|  |  |  |  |  |  |  |

8. Do you take your child to hospital if he got sick during teething?

| Yes | No |
| --- | --- |
|  |  |

9. What do you usually give your child when he starts teething?

| PARACETAMOL | Rubbing gum with garlic | Giving pacifier | HERBs | SALT WATER | Extracting the teeth | ANY OTHER |
| --- | --- | --- | --- | --- | --- | --- |
|  |  |  |  |  |  |  |

10. Who gave you the information on how to handle teething in babies?

| MOTHER | GRAND MOTHER | FRIENDS | FROM OBSERVATION | NURSE | DOCTOR |
| --- | --- | --- | --- | --- | --- |
|  |  |  |  |  |  |

11. What do you think will happen if you do not give the baby anything for teething?

| POOR GROWTH | SEVERE  ILLNESS | NOTHING | DEATH | ANY OTHER |
| --- | --- | --- | --- | --- |
|  |  |  |  |  |

12. Have you ever seen any child die from teething problems?

| YES | NO | HEARD STORIES | ONLY WARNED |
| --- | --- | --- | --- |
|  |  |  |  |

13. Did teething have any effects on the older children?

| YES | NO | DON’T KNOW |
| --- | --- | --- |
|  |  |  |

THANK YOU
